# Echoes of fluid spin

**DOI:** 10.1093/nsr/nwz153

**Published:** 2019-10-12

**Authors:** Huifeng Du, Chu Ma, Nicholas X Fang

**Affiliations:** Department of Mechanical Engineering, Massachusetts Institute of Technology, USA

Spin—an intrinsic angular momentum carried by elementary particles—has long been thought to be exclusive to quantum mechanical systems. The spin of photons, for example, is associated with the vectorial nature of electromagnetic waves [1]. In classical systems, however, the concept of a spinning degree of freedom is not as well defined. For instance, sound is often considered to be spinless due to the longitudinal and scalar-pressure field description of acoustic waves.

It comes down to the question of whether the scalar theoretical framework is a complete description of acoustics in some scenarios. Fundamentally speaking, vector field theory is required for the accurate depiction of hydrodynamics, and some information about the degree of freedom of microscopic fluid motion might be lost when using the scalar-pressure acoustic equations. Indeed, most of the time, the vector potential in Stokes flows is regarded as an arbitrary quantity that does not participate in the sound emission and momentum transfer. Recently, researchers from the University of California, Berkeley and Tongji University observed the spinning motion of fluids in sound propagation for the first time [2], with brand new physical insights of such vector potentials. They also observed acoustic-spin-induced torque due to the absorption of spin angular momentum on an acoustic probe, as well as spin-momentum locking.

**Figure 1.**
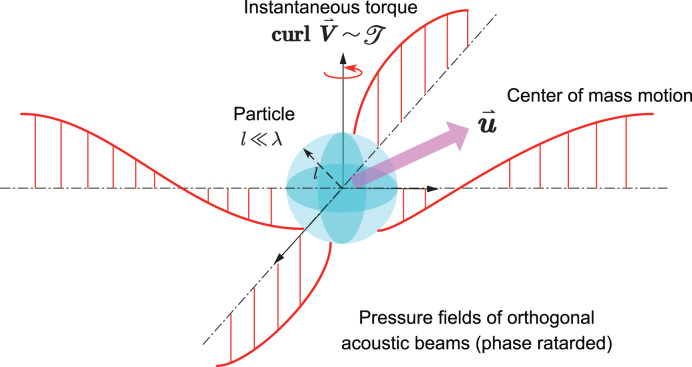
Illustration of fluid element motion decomposition and exerted torque on a spinnable particle. At the intersection of two orthogonal acoustic beams with retarded phases, the spinning fluid applies a torque on the object via angular-momentum transfer.

Scientists have long been seeking to establish a connection between quantum mechanical wave functions and fluid dynamics. For example, a set of well-established Madelung equations that illustrate the link between spin and orbital angular momentums [3] can be obtained via the decomposition of local velocity into two parts, as shown in Fig. 1: homogeneous component representing the velocity of the center of mass, plus the inhomogeneous and microscopic contribution, which is the velocity of motion in the center-of-mass frame (some named it the internal ‘spin motion’ or *Zitterbewegung*, ‘trembling motion’ in German). Furthermore, it has been demonstrated that the concept of Bohm quantum potential in the Madelung fluid model is an equivalent way of showing the existence of spin and *Zitterbewegung* of particles, thus providing a physical interpretation of the non-classical potential. For a given fluid flow at position *x*,

(1) \begin{eqnarray*} && {\boldsymbol x} \, \equiv\, {\boldsymbol \xi + X}\,\,\quad{\dot {\boldsymbol x}} \equiv {\boldsymbol \upsilon} = {\boldsymbol u} + {\boldsymbol V}\nonumber\\ &=& {\boldsymbol {p}_m} + \frac{{{\boldsymbol \nabla} \times (\rho {\boldsymbol s})}}{\rho}, \end{eqnarray*}

where ${\boldsymbol \xi} $ and ${\boldsymbol u} = \boldsymbol{\dot\xi} $ are the motion and velocity of the center of mass, and ***X*** and ***V*** ≡ ${\dot{\boldsymbol X}}$ represent the internal motion relative to the center-of-mass frame. The last equality was proposed by Hestenes in the absence of external electromagnetic vector potential [4]. Note the momentum in (1) is actually momentum density with respect to unit mass, and the same holds for the spin term. Since the curl of the gradient vanishes, the local rotational properties of the Madelung fluid can be shown to be [4]:

(2) \begin{eqnarray*} &&{\rm {rot}}{\boldsymbol\upsilon} = {\rm curl}{{\boldsymbol p}_m} + {\rm curl}{\boldsymbol V} = {\rm curl}{\boldsymbol \nabla} \varphi \nonumber\\ &&+\, {\rm curl}\,{\boldsymbol V} =\left[{{{\left({\frac{{\nabla \rho}}{\rho}} \right)}^2} - \frac{{\Delta \rho }}{\rho }} \right]{\boldsymbol s}\propto \mathcal{J}. \nonumber\\ \end{eqnarray*}

**Table 1.** Decomposition of fluid motion into center-of-mass motion in the classical sense and non-classical spin motion.

	Kinematics	Hydrodynamics	Quantum mechanics
Center-of-mass motion	***u***	${\mathit {rot}}\,{{\boldsymbol{p}}_m} = {\mathit {rot}}\nabla \varphi = 0$	$E = \frac{1}{2}m{{\boldsymbol{u}}^2}$ (kinetic energy)
Spin or *Zitterbewegung*	***V***	${\mathit {rot}}\frac{{\nabla \times (\rho s)}}{\rho }\propto \mathcal{J} $	$\frac{{{\hbar ^2}}}{{4m}}[ {\frac{1}{2}{{( {\frac{{\nabla \rho }}{\rho }} )}^2} - \frac{{\Delta \rho }}{\rho }} ]$ (Bohm Potential)

Wave function $\psi \equiv \sqrt {\rho} {{e^{i\frac{\varphi }{\hbar }}}\chi } $. Linear momentum and spin vector can be expressed accordingly with Pauli matrices ${\boldsymbol\sigma}\!: {\boldsymbol{p}} \equiv {\rho ^{ - 1}}\frac{{i\hbar }}{2}[ {( {\nabla {\psi ^\dagger }} )\psi - {\psi ^\dagger }\nabla \psi } ]$, $s \equiv {\rho ^{ - 1}}{\psi ^\dagger }\hat{\boldsymbol s}\psi $, $ \hat{\boldsymbol s} \equiv \frac{\hbar }{2}({\sigma _x};{\sigma _y};{\sigma _z})$.

One would immediately recognize that the second-to-last term is analogous to the Bohm potential in quantum mechanics, which reflects the non-classical spinning nature of the fluid motion, while the classical center-of-mass momentum ${{\boldsymbol p}_m} = \nabla \varphi $ vanishes with the curl operator (shown in Table 1). The last equation suggests that the angular-momentum transfer in fluids could, in principle, serve as an indicator of the spin vector. This provides a feasible route to deduce the acoustic spin by experimentally measuring the torque $\mathcal{J} $ exerted on a spinning object (2). The spin angular-momentum density *s* reflects the fluid's intrinsic property and is origin-independent; this is in contrast to the orbital angular momentum, which generally requires a reference point to define: ${\boldsymbol L} = \rho ({\boldsymbol r} \times {{\boldsymbol p}_m})$, where *r* is the position vector and the total angular momentum admits the following decomposition:

(3) \begin{equation*} {\boldsymbol J} = {\boldsymbol L} + {\boldsymbol S} = \rho ({\boldsymbol r} \times {{\boldsymbol p}_m}) + \rho {\boldsymbol s}.\end{equation*}

This implies that acoustic vortex beams are eigenmodes of the total angular-momentum operator instead of $\hat{L}$ or $\hat{S}$ separately.

In summary, Shi *et al.* offered new physical insights into the spinning-vector potential by evaluating the rotation angle on a coiled space meta-atom serving as an acoustic probe [2]. In addition, spin-momentum locking is revealed by showing the coupling of the spin-up or spin-down acoustic wave with a metamaterial waveguide composed of periodical grooves. The work provides the first demonstration of the existence of acoustic spin and opens the door for future explorations of the acoustic analogy of spin physics in quantum physics and electromagnetics, such as the topological transport of sound facilitated by the spin-redirection geometric phase [5]. It also offers a new degree of freedom to manipulate acoustic waves for applications of acoustic sensing or particle manipulation in complex fluid and soft condensed matters.

***Conflict of interest statement***. None declared.
